# Identification of Mobile Phones Using the Built-In Magnetometers Stimulated by Motion Patterns

**DOI:** 10.3390/s17040783

**Published:** 2017-04-06

**Authors:** Gianmarco Baldini, Franc Dimc, Roman Kamnik, Gary Steri, Raimondo Giuliani, Claudio Gentile

**Affiliations:** 1 European Commission, Joint Research Centre, Ispra 21027, Italy; gary.steri@jrc.ec.europa.eu (G.S.); raimondo.giuliani@jrc.ec.europa.eu (R.G.); 2 DiSTA, University of Insubria, Varese 21100, Italy; claudio.gentile@uninsubria.it; 3 Faculty of Maritime Studies and Transport, University of Ljubljana, Portorož 6320, Slovenia; franc.dimc@fpp.uni-lj.si; 4 Faculty of Electrical Engineering, University of Ljubljana, Ljubljana SI 1000, Slovenia; kamnikr@robo.fe.uni-lj.si

**Keywords:** fingerprinting, magnetometers, mobile phone

## Abstract

We investigate the identification of mobile phones through their built-in magnetometers. These electronic components have started to be widely deployed in mass market phones in recent years, and they can be exploited to uniquely identify mobile phones due their physical differences, which appear in the digital output generated by them. This is similar to approaches reported in the literature for other components of the mobile phone, including the digital camera, the microphones or their RF transmission components. In this paper, the identification is performed through an inexpensive device made up of a platform that rotates the mobile phone under test and a fixed magnet positioned on the edge of the rotating platform. When the mobile phone passes in front of the fixed magnet, the built-in magnetometer is stimulated, and its digital output is recorded and analyzed. For each mobile phone, the experiment is repeated over six different days to ensure consistency in the results. A total of 10 phones of different brands and models or of the same model were used in our experiment. The digital output from the magnetometers is synchronized and correlated, and statistical features are extracted to generate a fingerprint of the built-in magnetometer and, consequently, of the mobile phone. A SVM machine learning algorithm is used to classify the mobile phones on the basis of the extracted statistical features. Our results show that inter-model classification (i.e., different models and brands classification) is possible with great accuracy, but intra-model (i.e., phones with different serial numbers and same model) classification is more challenging, the resulting accuracy being just slightly above random choice.

## 1. Introduction

The identification of mobile phones through their built-in components has been extensively investigated by researchers for different electronic components: the internal digital camera [1], the RF transmission components for various communication standards (e.g., GSM, WiFi) as described in [2,3], the microphones [4,5] and the accelerometers [6,7]. The identification is performed by exploiting tiny physical differences, which characterize electronic components due to the manufacturing process or the use of different materials. These differences can be observed in the digital output generated by the electronic components when they are stimulated by a similar or identical input (e.g., motion pattern stimulus to an accelerometer). Through a statistical analysis of the digital output, it is possible to extract the fingerprints and use machine learning algorithms to classify and identify the devices (e.g., mobile phones). The proposed classification and identification of the devices is based on the fact that physical differences impact the statistical features of the digital output (e.g., variance, kurtosis). Because it is not known a priori which statistical features are more relevant, experimental campaigns are needed in order to identify the most appropriate statistical features.

The identification of mobile phones on the basis of their physical features is an important function in many applications. For example, the correct identification of electronic devices (e.g., mobile phones) is useful in the fight against the counterfeiting and distribution of electronic goods. Many tools and methods to identify counterfeit products are based on visual or augmented inspection techniques, like Scanning Acoustic Microscopy (SAM) and Scanning Electron Microscopy (SEM). Since these methods have an important drawback of requiring expensive tools, some researchers have instead proposed more cost-effective approaches based on the statistical analysis of the digital output of the components [8].

Another application is related to multi-factor authentication, where the physical properties of the mobile phone can complement or augment authentication methods based on cryptographic means, that is the identification based on a cryptographic key can be combined with the identification based on the physical properties. This approach was used in [9,10].

The identification of mobile phones on the basis of their built-in components, like RF transmission components, digital cameras, microphones and accelerometers, has been extensively investigated in the literature. However, no cases on mobile phone identification based on magnetometers have been reported to date. A survey of the different techniques and approaches is presented here.

Mobile phone identification based on the RF fingerprinting has been applied to many different wireless communication standards supported by the mobile phone, like WiFi, GSM, Bluetooth, and so on. In most cases, the RF fingerprinting is implemented by collecting the RF emissions when the mobile phone is transmitting. For example, in the case of GSM or WiFi, the mobile phone emits bursts of traffic, which are repeated with a high frequency. An RF receiver (e.g., a spectrum analyzer or a digitizer) is used to collect the signal in space, downsample it, filter it and then digitize it. The physical differences of the RF transmitter appear in the time series of the digitized collected bursts as small variations between one mobile phone to another. Then, specific statistical features like variance, skewness and kurtosis are extracted from the time series and used as mobile phone fingerprints. This is the approach used for WiFi in [11] and for GSM in [12].

A similar approach is based on the processing of the digital output generated by the sensors present in the mobile phone. By following this approach, the observables used for the mobile phone identification are not collected by an external device, but they are collected by the mobile phone itself once the sensors are stimulated in some way. This approach has been used for the internal digital cameras starting from the pioneering work by Lukas et al. [1]. In a digital camera, several sources of imperfection and noise show up during the various stages of the image acquisition process. Even if the imaging sensor takes a picture of an absolutely uniformly lit scene, the resulting digital image can exhibit small changes in intensity between individual pixels. These changes are due to various hardware imperfections or differences among cameras, which include lens radial distortion, chromatic aberrations, dust on the lens, sensor pattern noise, high-ISO sensitivity noise, white noise and shot noise. Some of these noise components (e.g., high-ISO noise, white noise and shot noise) have a random distribution, and if a large number of frames are used and added or averaged together, these noise components tend to cancel out. This is the case of SPN, which has been widely used to uniquely identify with high accuracy digital cameras and mobile phones, as described in the survey paper [13]. In this case, the stimulation is the light received by the camera.

Fingerprinting through the built-in microphones characterization of a mobile phone was also investigated in the literature by [5,14,15,16]. The verification and identification of mobile phones with audio acquisition capability (e.g., mobile phones, tablets, webcams, camcorders, cordless phones) can be achieved by analyzing the response of the audio circuit to a standard stimulus (e.g., a standard tone). Because of the nominal values of the electronic components and the different designs employed by the various manufacturers, the microphones of the different mobile phones introduce a different convolution distortion of the input audio signal (i.e., frequency response), which becomes part of the recorded audio. In general, the authors of the above papers use the MFCC to define the features used for fingerprinting as commonly employed to fingerprint human speakers. Most of the papers use SVMs to classify mobile phones on the basis of the audio recordings.

Mobile identification based on MEMS sensor fingerprinting and, in particular, on accelerometers has been presented mainly in [6,17,18] where the authors describe the experimental identification of mobile phones using their built-in accelerometers and gyroscopes. Data are collected when the phones are subject to repeatable movements performed by a high precision robotic arm, so that a considerable dataset from which are extracted several statistical features is obtained. Then, using an SVM classifier, phones of the same brand and model are identified with an accuracy higher than 90% for some combination of features. Usually, the authors use variance, skewness, kurtosis and entropy-related (e.g., Shannon entropy, log entropy, threshold entropy) features for classification. Results show that, if properly stimulated, built-in accelerometers and gyroscopes can be used to extract fingerprints that allow for a very precise intra-model identification, thus confirming the applicability to anti-counterfeiting and other scenarios.

To our knowledge, no authors have attempted to identify and classify mobile phones on the basis of the built-in magnetometers, which are subject to a motion pattern.

The objective of this paper is to evaluate a technique for mobile phone identification based on the built-in magnetometers of the mobile phone, which are now present in most of the recent models of mobile phones. The technique is based on the stimulation of the magnetometers using a rotating platform with a fixed magnet. A mobile phone is installed on a cost-effective rotating platform spinning at a constant speed. Every time the mobile phone passes in front of the magnet, the magnetic field stimulates the magnetometer of the mobile phone. The digital output of the magnetometer is collected by the mobile phone itself and processed through appropriate statistical tools. In particular, statistical features like variance, skewness and kurtosis are extracted and used as fingerprints. A SVM learning algorithm is used to classify the different mobile phones on the basis of the extracted statistical features. SVMs are used here for their superior performance to other machine learning algorithms, like KNN and naive Bayes. This difference in performance among the machine learning algorithms is reported in the Results section of this paper. For each mobile phone, the experiment is repeated across six different days to ensure consistency in the results. A total of 10 phones from different brands and models or of the same model were used in the experiment. Our experimental evidence shows that inter-model (i.e., different models and brands) classification is possible with great accuracy, but intra-model (i.e., phones with different serial numbers and same model) classification is far more challenging, the resulting accuracy being just slightly better than random guessing.

The remainder of the paper is organized as follows: Section 2 provides the overall methodology for the fingerprinting data collection, analysis and comparison. Section 3 shows the results of our tests, while in Section 4, we wrap-up, make final comments and point to future work.

## 2. Methodology for Data Acquisition and Processing

**Figure 1:**
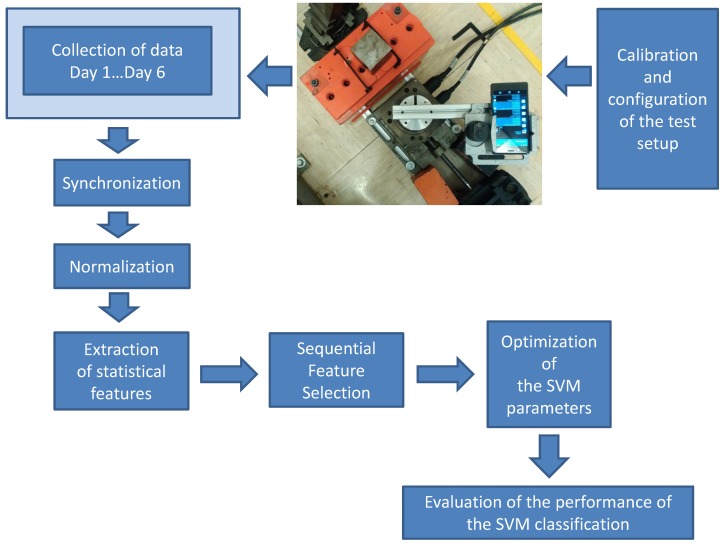
Methodology used to classify mobile phones using the built-in magnetometers.

The overall methodology flow used in the paper for the collection of data, processing and analysis is shown in Figure 1. Each step is described in the following paragraphs.

**Figure 2:**
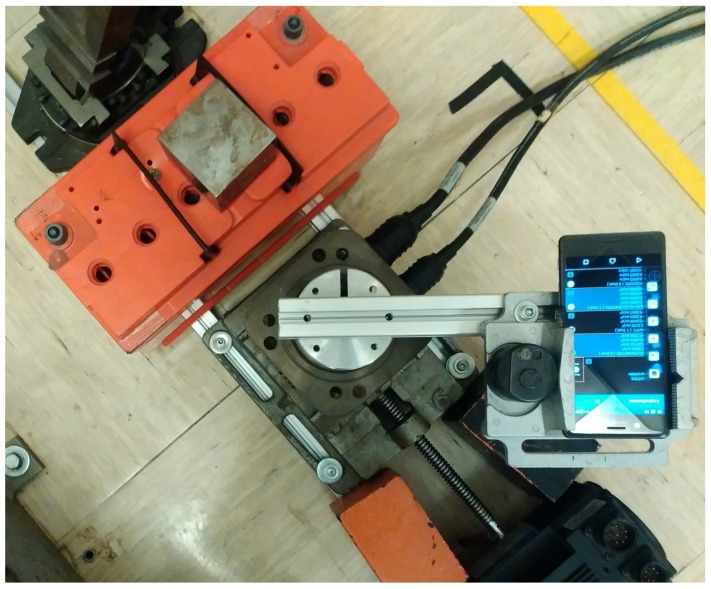
Image of the test setup used to collect the data.

The initial step is the setup of the test bed where the rotating platform for the definition of the motion pattern is configured. The test bed is illustrated in Figure 2, where a mobile phone is installed on a cost-effective rotating platform and a magnetic element (an iron cube) is positioned at one extreme of the test bed. The rotating platform rotates the mobile phone with a specific motion pattern. The built-in magnetometer is stimulated by the magnetic element when it passes over it. The magnetic perturbation is collected and analyzed using an Android application installed in the mobile phone. In this experiment, we have used the AndroSensor application, but any other application that is able to record the digital output from the magnetometer can be used.

The application was configured to record the magnetometer digital output with a sampling time of 0.05 s. The motion pattern used in our experiment was as follows: +120 rpm then −120 rpm for 4 s, +150 rpm then −150 rpm for 3 s, +180 rpm then −180 rpm for 2 s. Each mobile phone was kept for 60 s before the start of the motion pattern in a fixed position in front of the magnet. Each mobile phone was subject to this motion pattern. A total of 10 mobile phones was used in the experiments. Table 1 shows the brand and models of the phones used in the experiment. We note that three phones were from the same brand and model (i.e., HTC One X), while the other phones were from different brands and models.

In each measurement campaign, each mobile phone is subject to 25 repetitions of the motion pattern. This experimental campaign was executed during six different days (even at the distance of a week), so as to ensure that the fingerprints are stable over time. As a consequence, we have a total of 25*6 = 150 motion patterns (henceforth called responses in the rest of this paper), which can be used for classification.

**Figure 3:**
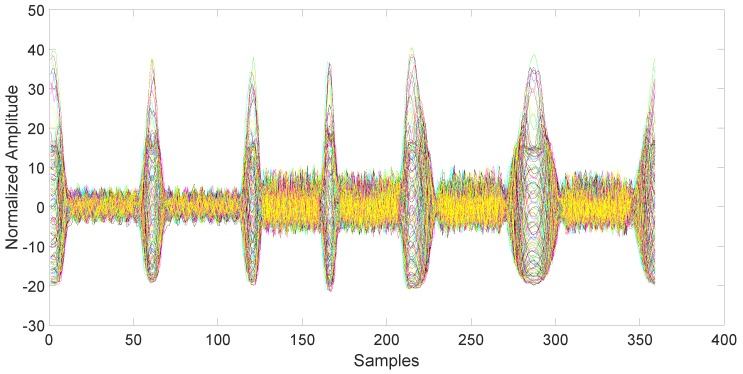
Magnetometers output for all 6 days of data collection after synchronization and normalization. Each color represents a different day.

**Figure 4:**
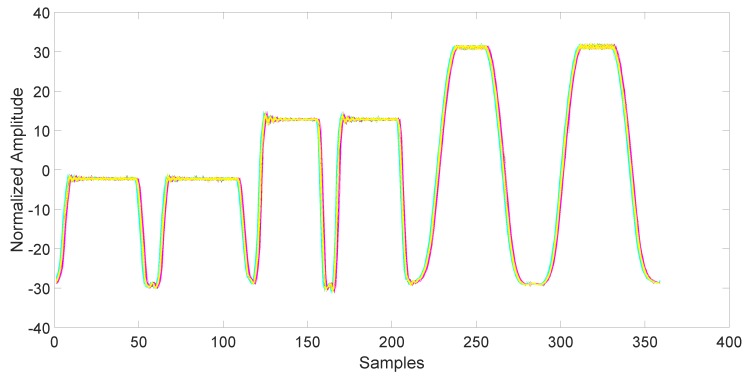
Accelerometers output for all 6 days of data after synchronization and normalization. It has been used for the synchronization of the responses. Each color represents a different day.

After collection, the data must be synchronized and normalized. This is an important step, since unsynchronized/unnormalized data can introduce a severe bias in the classification. Since the data collected by the magnetometers are particularly noisy (see Figure 3), the synchronization is done using the related accelerometers data, which are also collected by the AndroSensor application with the same rate (see Figure 4). The synchronization is performed using the variance trajectory technique. This technique is based on the calculation of the variance on a sliding window of samples, which moves along the response. The variance will increase substantially when the sliding windows meet a sharp rise or fall of the response. The rise of the variance identifies the beginning and the end of the response. This process is applied to all 150 responses gathered in the collection phase. The application of variance trajectory was inspired by its use in RF fingerprinting to detect the start and end of the wireless communication bursts [11]. After synchronization, the data are normalized. The normalization is carried out by applying the RMS to each single response for each individual mobile phone.

To ensure that the fingerprints are stable over time, the classification through machine learning tools (described later on) is performed on the combination of the 150 collected responses. In other words, the representative set of each phone for classification is made up of 150 responses.

The next step is to extract the statistical features from the 150 responses, which can be seen as time series with specific characteristics of variance or entropy. We follow a similar approach as those proposed in the literature for different built-in components (e.g., RF and accelerometers), where variance, skewness, kurtosis and entropy are calculated for each response. Table 2 shows the set of statistical features used in our classification problem.

Now, since the resulting set of features is large, it is important to identify the subset of features that are expected to provide the best identification and verification accuracy. The process to achieve this goal is called feature selection. Various approaches to feature selection have been proposed in the literature (see, e.g., [19]). In this paper, we combine the SFS algorithm with a brute force approach. SFS starts with a single feature or a small set of features and incrementally adds a new feature at the time by measuring the resulting value of a given metric. If the metric improves, the feature is added; otherwise, another feature is checked for inclusion. The process continues until no further improvement of the metric is detected.

In this paper, a metric based on the overall accuracy of the confusion matrix was used for the SFS algorithm. Moreover, in order to avoid local maxima, a brute force search was also performed to select one or a few sets of combinations of 4 features among all possible combinations (sets of 4 features out of 18, which results in 184=3060 sets of features to check). In the brute force approach, all possible combinations of the 4 features were calculated. Then, the best combination of the 4 features was selected to seed the SFS algorithm, which computed the remaining features to add.

Once the best set of features is selected, the parameters of the machine learning algorithm at hand must be optimized. The execution of SFS is already based on optimal values reported in the literature for the application of SVM to fingerprinting. Yet, since it is the first time that classification of mobile phones based on magnetometers is attempted, the optimization of the parameters is performed specifically for the collected set of responses. As described in Section 3, a 3-fold approach was used for classification based on machine learning tools, and this process was repeated 50 times. For each repetition and each fold, feature selection and optimization of parameters is performed on the training set only, and classification accuracy is computed only on the test set. The histograms of the recurrence of the selected features, as well as the optimal values of parameters are provided in Section 3.

The final step is the classification itself, which is done through SVMs, widely adopted in fingerprinting (see [5,6,20]). A comparison with other standard classifiers (KNN, naive Bayes and random forests) is also carried out and reported.

In standard machine learning classification settings, classification performance is measured as follows. A given class is taken as a reference class (usually called the “positive” class), then the following quantities are computed:

- $T_{p}$ is the number of true positive matches, where the machine learning algorithm has correctly identified a sample (e.g., a collected RF signal in our context) as belonging to the positive class;
- $T_{n}$ is the number of true negative matches, where the machine learning algorithm has correctly identified a sample as not belonging to the positive class;
- $F_{p}$ is the number of false positive matches, where the machine learning algorithm has mistakenly identified a sample as belonging to the positive class;
- $F_{n}$ is the number of false negative matches, where the machine learning algorithm has mistakenly identified a sample as not belonging to the positive class.

One of the standard adopted metrics is the accuracy, which is defined as:

(1) $$Accuracy=\frac{T_{p}+T_{n}}{T_{p}+T_{n}+F_{p}+F_{n}},$$

where $T_{p}$ is the number of true positives and $T_{n}$ is the number of true negatives resulting from the application of the SVM machine learning algorithm to the problem of verifying that the collected fingerprints are representative of the same magnetometer evaluated in the training phase (i.e., for verification).

The ROC is generated by plotting the $T_{p}$ rate vs. $F_{p}$ rate in a binary classifier system as its discrimination threshold is varied.

The EER corresponds to the condition on the ROC curve where $T_{p}$ and $F_{p}$ are equal. In this paper, the value of the EER is calculated for the X-axis. This metric is frequently used as a summary statistic to compare the performance of various classification systems. In general, the lower EER, the better the classification performance.

Finally, the confusion matrix is also used to show the results of the identification process. In the confusion matrix, each column of the matrix represents a predicted class, while each row represents the actual class. As in our experiments we used 10 phones, the confusion matrix has a dimension of 10 × 10. In the confusion matrix, the correct guesses (i.e., true positive or negative) are located in the diagonal of the table, so it is easy to inspect the table for errors, as they will be represented by values outside the diagonal. The overall accuracy can be defined as the sum of the elements on the diagonal over the total sum (which in our case, equals 1500, i.e., 150 responses for 10 phones).

## 3. Experimental Results

### 3.1. Features and Parameters Optimization

In this section, we describe how the features are constructed and how parameter optimization is performed.

The features used in this paper are based on similar works cited in the Introduction: entropy-based features, variance, standard deviation, skewness and kurtosis. These features are applied both in the time domain and the frequency domain after a FFT is applied. Each feature is identified by the associated number as shown in Table 2.

The features can refer to each of the three axes of the magnetometers. Their selection has indeed been applied to all three axes.

As described in Section 2, the SFS algorithm is used in combination with a brute force approach. The metric used for the evaluation of the performance of the SFS algorithm is the overall accuracy of the confusion matrix derived from the application of a multiclass SVM. SVM is traditionally a binary classifier, so it must be combined with a multi-class approach to provide multiclass classification (as in our case, where we need to classify 10 mobile phones). In this paper, we will use the one-vs.-one approach, where for each binary learner, one class is positive, another is negative and the remaining classes are ignored. This approach exhausts all K2=K(K−1)/2 combinations of class pair assignments. One-vs.-one is much less sensitive to the problems of imbalanced datasets than alternative approaches like one-vs.-all, but on the other hand, is more computationally expensive (one-vs.-all trains *K* classifiers only) [21]. Because we have a limited set of responses for each phone (i.e., 150) and the computational performance is not an issue, we selected the one-vs.-one approach. The ratio of the diagonal elements of the confusion matrix to the sum of all of the elements of the confusion matrix is the overall accuracy, which is the metric used in the SFS algorithm. Note that the overall accuracy includes both intra-model and inter-model accuracy because in our experiment, the set of phones includes both different models and the three phones of the same model (the three HTC phones).

Classification performance is evaluated using three-fold cross-validation. Each collection of statistical fingerprints (one for each mobile phone) is divided into three blocks, each having 50 fingerprints per block. Two blocks from each device are used for training, and one block is held out for classification. The training and classification process is repeated three times until each of the three blocks has been held out and classified. Thus, each block of statistical fingerprints is used once for classification and twice for training. Final cross-validation performance statistics are calculated by averaging the results over all folds.

As described in Section 2, the optimization process was repeated for each of the three folds on the training set only. Finally, the overall process was repeated 50 times. While, this can be a time-consuming process, it mitigates the risk of high variance in the results and provides a good evaluation of the relevance of the statistical features.

**Figure 5:**
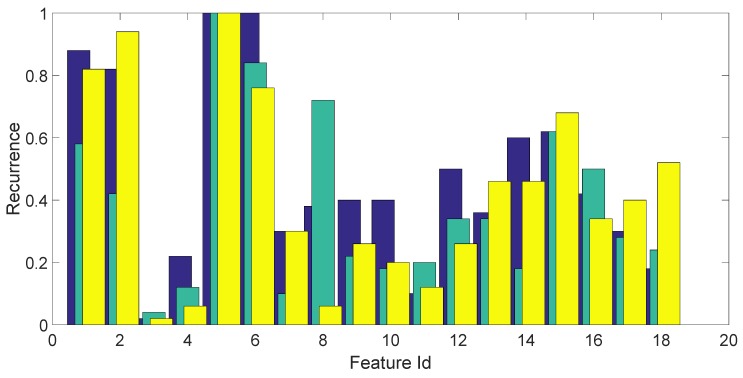
Frequency of occurrence of the 18 statistical features on the three folds, averaged over the 50 repetitions of the feature selection process. The three folds are depicted in three different colors.

A bar chart showing the (average) fraction of times each feature gets selected for each fold is shown in Figure 5. Each fold is represented by a different color. This bar chart shows a predominance of entropy features, skewness and kurtosis in the time domain (Features 1, 2 and 5, 6), but also, the features in the frequency domain (amplitude) are somewhat relevant (Features from 13 to 18).

Further parameters have to be tuned depending on which machine learning algorithm we are adopting. As described in [21], the SVM algorithm must be optimized on the *C* parameter (the so-called box constraint parameter), allowing the SVM user to control the weight of the classification errors during training, and the kernel function, which is used to define the shape of the computed hyperplane. Various kernel functions are available in the literature including linear, polynomial and RBF. In this paper, we use SVM with RBF as a kernel function because this combination has demonstrated its effectiveness for fingerprinting classification in [20] and other references.

We recall that the definition of the RBF is the following:

(2) $$K\left(x_{i},x_{j}\right)=e^{-\gamma ∥x_{i}-x_{j}{∥}^{2}}\;,$$

where the scaling factor γ is the second parameter to be tuned together with the box constraint parameter *C*. Both *C* and γ are positive real values.

**Figure 6:**
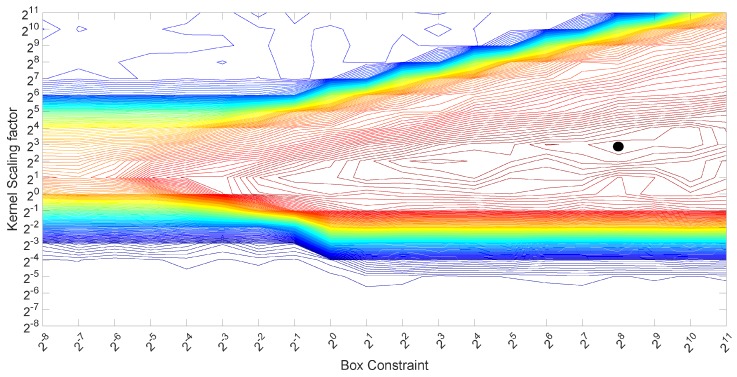
Grid (contour plots) for the optimization of the box constraint parameter *C* and the scaling factor γ for SVM.

Various techniques can be used to optimize these values. In this paper, we adopt the grid approach with a set of exhaustive exponential values to base two, from $2^{0}$ to $2^{6}$ for the scaling factor γ and from $2^{0}$ to $2^{11}$ for the box constraint parameter *C*. These range of values were based on a previous optimization process, which has shown that values outside these ranges provided a low classification accuracy. See Figure 6, which shows an example of the previous optimization process with an extended range of values. The optimal values in this example are highlighted with the black circle mark in the figure.

**Figure 7:**
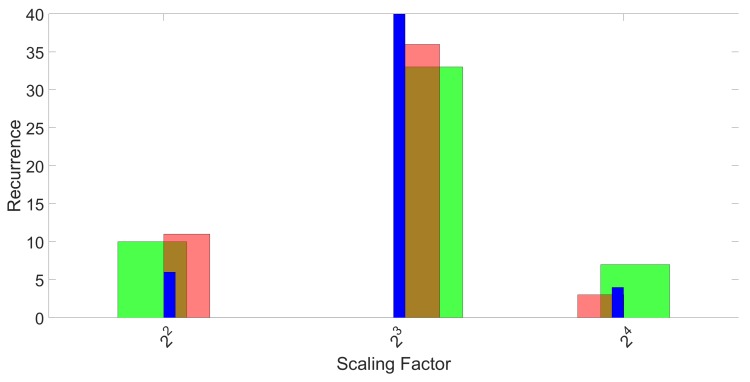
Histograms of the recurrence of the best performing scaling factor γ for 50 repetitions and three folds. The three colors represent the three folds.

**Figure 8:**
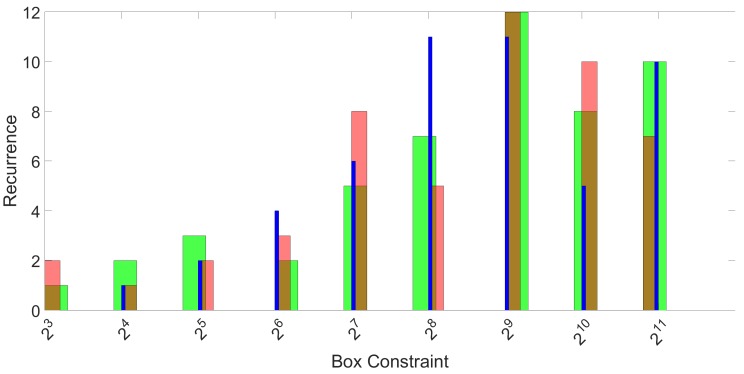
Histograms of the recurrence of the best performing box constraint parameter *C* for 50 repetitions and three folds. The three colors represent the three folds.

This process was repeated for all 50 repetitions and the three folds. The final result of the SVM parameter optimization effort is shown in Figure 7 for parameter γ and in Figure 8 for parameter *C*. Again, the three different colors represent the three different folds.

On the basis of the selected features, and the identified optimal values for *C* and γ, we can run the classification on the test set (held out folder) and analyze the results we obtain. This is reported in the next section.

### 3.2. Classification Results

The confusion matrices obtained in SVM classification, after training and tuning parameters as described in the previous section, are shown in Table 3, Table 4 and Table 5, which correspond to responses along the three axes of the magnetometers.

From Table 3, we conclude that classification accuracy is quite high for mobile phones of different brands and models, but it is much lower (almost getting to random choice) for mobile phones of the same model and different serial numbers.

For the sake of completeness, we have carried out a comparison of different machine learning classification algorithms to check whether SVM has indeed a superior performance.

Table 6 reports comparative performance when operating on responses along the *X* axis, similar comparative results hold for axes *Y* and *Z*. The other algorithms were also optimized on the basis of their specific parameters (e.g., the number of neighbors for KNN, prior parameters for naive Bayes, the number of decision splits for classification trees).

From Table 6, we can clearly see that SVM offers a superior classification performance as compared to standard baselines for this particular classification problem.

The results presented so far were based on the digital output gathered from the magnetometer in the *X* direction. We now evaluate and compare classification performances obtained with the digital output taken from the magnetometer along the *Y* axis (Figure 4) and the *Z* axis (Figure 5).

From the different confusion matrices, one can see that the accuracy pattern is similar for all three axes (high inter-model accuracy, but low intra-model accuracy). The overall accuracy for classification based on the *Z* axis is 81.46% (the ratio of the sum over diagonal values of the confusion matrix in Table 5 to the sum over off-diagonal values), while the overall accuracy based on the *Y* axis is 76.02%, and the overall accuracy for the *X* axis is 70.61%.

**Figure 9:**
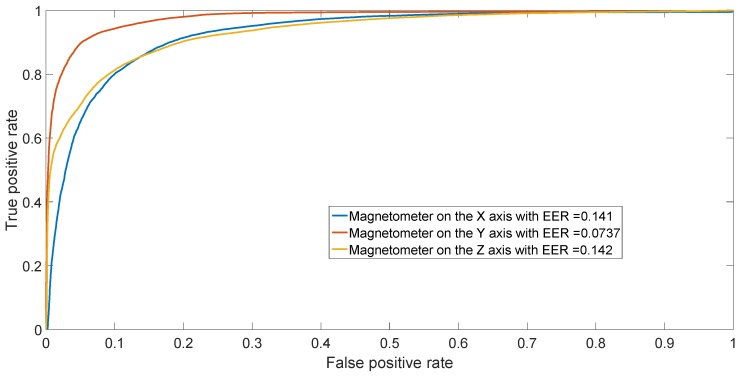
ROC achieved by SVM in binary classification between Sony Experia X and Samsung Galaxy S7. Results have been averaged over the 50 repetitions.

**Figure 10:**
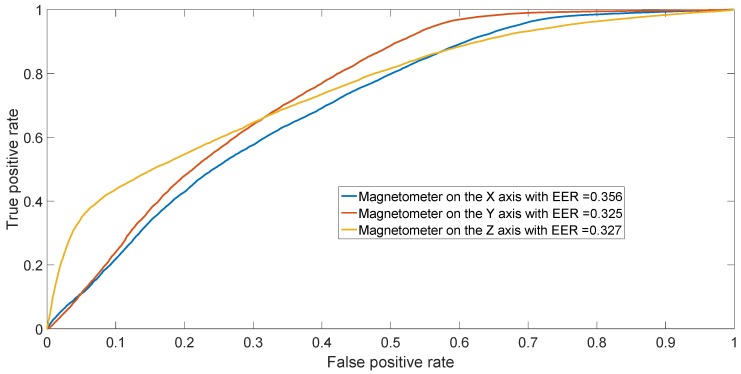
ROC achieved by SVM in binary classification between HTC One X 2 and HTC One X 3. Results have been averaged over the 50 repetitions.

The results from the confusion matrices can also be confirmed by performing binary classification separating two phones of different models (inter-model classification) and two phones of the same model (intra-model classification). The resulting ROCs are depicted in Figure 9 for different models (Sony Xperia X vs. Samsung Galaxy S7) and in Figure 10 for two HTC One X mobile phones (HTC One X 2 vs. HTC One X 3). The figures illustrate the ROCs for all three different axes of the magnetometers, averaging the results across the 50 repetitions.

We complement the previous results by reporting the average inter-model and intra-model identification accuracy for all three axes of the magnetometers in Table 7. The inter-model accuracy is calculated as the average classification accuracy when including only one HTC mobile phone (i.e., phone identifiers from 1 to 8). The intra-model accuracy is computed when operating only with the three HTC mobile phones (i.e., phone identifiers from 8 to 10).

### 3.3. Addition of Gaussian Noise

In a practical application of mobile phone identification based on the fingerprints of the built-in magnetometers, it is well possible that the distance between the mobile phone and the magnetic element stimulating the magnetometer can vary. Changes in distance and orientation will definitely impact the SNR. Different distances and different values of SNR can be simulated by adding AWGN to the collected magnetometers responses.

**Figure 11:**
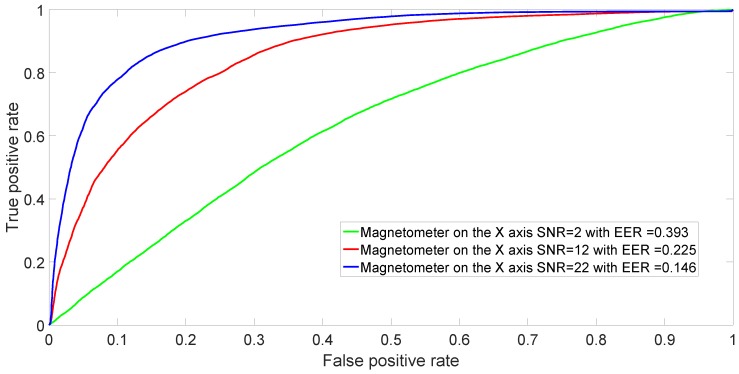
ROC achieved by SVM in binary classification between Sony Xperia X and Samsung Galaxy S7 using the *X*-axis for decreasing values of SNR. Again, these curves are obtained after averaging over 50 repetitions.

Figure 11 shows the ROCs for binary classification between Sony Xperia X and Samsung Galaxy S7 for decreasing values of SNR. The associated value of the EER is shown in the caption. As expected, a low value of SNR results in almost random choice identification (e.g., the green curve) because the machine learning algorithm is not able to leverage very noisy signals.

### 3.4. Combination of Features from Different Magnetometers to Improve Accuracy

As a final step, we have attempted to combine the responses along all of the three axes to improve identification accuracy. Our experimental findings show that, despite the fact that the overall processing time is longer, significant improvements in the overall accuracy can be achieved.

The best set of features from all three axes has been combined into a single matrix fed to the SVM algorithm. The resulting confusion matrix is shown in Table 8. Since the set of features is larger (18 features per 3 axes = 54 features), the brute force approach was not used in the optimization phase. In its stead, the best set of features from each axis was used as the seed for the SFS algorithm to obtain the best set of features in each fold and in each iteration. The optimization of the box constraint and scaling factor parameters was implemented as for the single axis case.

The resulting overall accuracy is 85.08%, with an inter-model accuracy of 98.07% and an intra-model accuracy of 54.15%. These figures are higher than considering each axis in isolation. Specifically, there is a significant improvement (almost 4%) for inter-model accuracy, as compared to the best result of the single axis (magnetometer in the *Z* direction; see Table 7) and a slight improvement in intra-model accuracy.

## 4. Conclusions

In this paper, we have described a potential approach for the practical identification of mobile phones using their built-in magnetometers stimulated through a motion pattern and a magnetic element. The motion pattern is implemented with a simple rotating platform, which moves the mobile phone over the magnetic element. The experiment has been carried out over six different days to prove the stability of the fingerprints. The testbed where the measurements has been performed was not ideal for our purpose. The magnetic fields generated by the arm electric motor and the nearby motors used to generate the motion pattern are not shielded, and other ferromagnetic objects are also present in the laboratory where data have been gathered. The SVM machine learning algorithm has been used in this experiment for mobile phone classification, and its superior performance in comparison to other machine learning classifiers has been shown. The responses taken along all three axes of the built-in magnetometers have been used and compared. The *Z*-axis provides a slightly better accuracy compared to the other two axes. In the final classification experiment, all three axes have been used for classification, yielding significantly improved performance. The resulting classification accuracy is quite high for inter-model classification (it reaches 98.07% when using all three axes), but is relatively poor for intra-model classification (as low as 54.15% when using all three axes). Different reasons can be put forward to explain the low intra-model classification: from the noisy environment to the small number of samples in the response, but also the location of the sensor in the mobile phone (i.e., in different models, the magnetometer might be placed in different positions, so that the fingerprint of the overall system smartphone-sensor is more distinctive). The small number of samples could be increased by lowering the speed of the motion pattern, but this has the obvious drawback that the experiment would take longer. The number of samples could also be increased by increasing the frequency of collecting the samples from the magnetometers, but older phones have a limit on the collection frequency (which is the one used in this paper). The authors will investigate alternative approaches to stimulate the built-in magnetometers of the mobile phones with the goal of generating improved fingerprints for both inter-model and intra-model classification.

## Figures and Tables

**Table 1:** List of mobile phones used in our experiment.

Mobile Phone Model	Number of Devices
Sony M4 Aqua	1
Huawei P8	1
Sony Xperia X	1
Samsung Galaxy S7	1
Huawei Mate 8	1
HTC One A9	1
LG G4	1
HTC One X	3

**Table 2:** Statistical features used for fingerprinting.

Feature Name	Time Domain	Frequency Domain (Phase)	Frequency Domain (Amplitude)
Shannon Entropy	1	7	13
Log Energy Entropy	2	8	14
Variance	3	9	15
Standard Deviation	4	10	16
Skewness	5	11	17
Kurtosis	6	12	18

**Table 3:** Confusion matrix based on SVM and optimal values for the digital output generated by the magnetometer on the *X* axis. Values have been averaged over the 50 repetitions.

	Sony M4	Huawei P8	Sony Xperia X	Samsung Galaxy S7	Huawei Mate 8	HTC A9	LG G4	HTC One X 1	HTC One X 2	HTC One X 3
**Sony M4**	49.053	0	0	0	0	0	0.02	0.113	0.54	0.293
**Huawei P8**	0	49.453	0	0	0	0	0.546	0	0	0
**Sony Xperia X**	0	0	30.126	1.613	12.526	5.733	0	0	0	0
**Samsung Galaxy S7**	0	0	2.606	33.16	9.3	4.933	0	0	0	0
**Huawei Mate 8**	0	0	10.253	6.36	30.813	2.573	0	0	0	0
**HTC A9**	0	0	6.08	1.513	2.76	39.646	0	0	0	0
**LG G4**	0	2.073	0	0	0	0	47.926	0	0	0
**HTC One X 1**	0	0	0	0	0	0	0	25.573	17.5	6.926
**HTC One X 2**	0	0	0	0	0	0	0	18.933	22.36	8.706
**HTC One X 3**	0	0	0	0	0	0	0.006	11.94	13.106	24.946

**Table 4:** Confusion matrix based on SVM and optimal values for the digital output generated by the magnetometer on the *Y* axis. Values have been averaged over the 50 repetitions.

	Sony M4	Huawei P8	Sony Xperia X	Samsung Galaxy S7	Huawei Mate 8	HTC A9	LG G4	HTC One X 1	HTC One X 2	HTC One X 3
**Sony M4**	49.153	0	0.093	0.306	0	0.093	0	0	0	0.353
**Huawei P8**	0	49.246	0	0	0	0	0.753	0	0	0
**Sony Xperia X**	0	0.02	43.413	2.7	3.866	0	0	0	0	0
**Samsung Galaxy S7**	0.02	0.113	3.146	43.58	2.733	0.226	0.08	0	0	0.1
**Huawei Mate 8**	0	0	6.12	2.566	31.18	10.133	0	0	0	0
**HTC A9**	0	0	0	0.033	6.52	43.446	0	0	0	0
**LG G4**	0.013	2.113	0	0.146	0	0	47.667	0	0	0.06
**HTC One X 1**	0	0	0	0	0	0	0.0267	23.006	21.04	5.926
**HTC One X 2**	0	0	0	0	0	0	0	20.826	25.033	4.14
**HTC One X 3**	0	0	0	0.06	0	0	0.053	14.506	11.0267	24.353

**Table 5:** Confusion matrix based on SVM and optimal values for the digital output generated by the magnetometer on the *Z* axis. Values have been averaged over the 50 repetitions.

	Sony M4	Huawei P8	Sony Xperia X	Samsung Galaxy S7	Huawei Mate 8	HTC A9	LG G4	HTC One X 1	HTC One X 2	HTC One X 3
**Sony M4**	49.333	0	0.013	0	0	0	0.34	0.113	0.08	0.12
**Huawei P8**	0	47.926	0.053	0	0	1.873	0.006	0.066	0	0.073
**Sony Xperia X**	0.006	0.306	41.28	5.293	0.733	0.32	2.06	0	0	0
**Samsung Galaxy S7**	0.106	0	6.053	43.66	0.18	0	0	0	0	0
**Huawei Mate** 8	0	0	0.84	1.18	47.98	0	0	0	0	0
**HTC A9**	0	0.786	0.12	0	0.006	48.426	0.313	0.026	0.06	0.26
**LG G4**	0.006	0.28	0.573	0	0	0.4	48.733	0	0.006	0
**HTC One X 1**	0	0.073	0	0	0	0.013	0	22.233	19.8	7.88
**HTC One X 2**	0.046	0	0	0	0	0	0	19.086	22.426	8.44
**HTC One X 3**	0.173	0	0.153	0	0	0.1	0	8.5	5.76	35.313

**Table 6:** Comparison among the different machine learning algorithms for the digital output generated by the magnetometer on the *X* axis. Accuracy values have been averaged over the 50 repetitions.

Machine Learning Algorithm	Overall Accuracy for X axis
SVM	70.61 %
KNN with number of neighbors = 3 (optimal value in a range from 1 to 10)	66.39 %
Naive Bayes with Gaussian kernel smoothing type (optimal kernel smoothing type [22])	62.98 %
Classification tree (optimized on maximal number of decision splits = 10 in a range from 2 to 20 and minimum observations per leaf = 3 in a range from 1 to 10)	65.58 %

**Table 7:** Average overall accuracy for inter-model and intra-model classification.

**Inter-Model**	
Magnetometer Axis	Overall Accuracy
*X*-axis	82.52%
*Y*-axis	89.35%
*Z*-axis	94.32%
**Intra-Model**	
Magnetometer Axis	Overall Accuracy
*X*-axis	48.37%
*Y*-axis	48.10%
*Z*-axis	53.5%

**Table 8:** Confusion matrix using all axes of the magnetometers.

	Sony M4	Huawei P8	Sony Xperia X	Samsung Galaxy S7	Huawei Mate 8	HTC A9	LG G4	HTC One X 1	HTC One X 2	HTC One X 3
**Sony M4**	49.286	0.013	0	0	0	0	0.493	0.120	0.08	0.006
**Huawei P8**	0	50	0	0	0	0	0	0	0	0
**Sony Xperia X**	0.0133	0.006	47.406	2.126	0.313	0	0.126	0.006	0	0
**Samsung Galaxy S7**	0.006	0.053	1.16	48.02	0.613	0.013	0.126	0	0.006	0
**Huawei Mate 8**	0	0	0.493	1.48	48.02	0.006	0	0	0	0
**HTC A9**	0	0	0	0.02	0	49.98	0	0	0	0
**LG G4**	0.013	0.1	0	0.006	0	0	49.866	0	0.0133	0
**HTC One X 1**	0	0	0	0	0	0	0.066	24.113	17.153	8.666
**HTC One X 2**	0	0	0	0	0	0	0	16.866	24.713	8.420
**HTC One X 3**	0	0	0	0	0	0	0	8.293	7.7	34.006

## Abbreviations

The following abbreviations are used in this manuscript:

AWGN	Additive White Gaussian noise
EER	Equal Error Rate
FFT	Fast Fourier Transform
GSM	Global System for Mobile Communications
IC	Integrated Circuit
KKT	Karush–Kuhn–Tucker
KNN	K-Nearest Neighbor
MEMS	Micro Electro-Mechanical Systems
MFCC	Mel-Frequency Cepstrum Coefficient
MLP	Multilayer Perceptron
PKI	Public Key Infrastructure
RBF	Radial Basis Function
RF	Radio Frequency
RMS	Root Mean Square
ROC	Receiver Operating Characteristics
SFS	Sequential Feature Selection
SMO	Sequential Minimal Optimization
SNR	Signal Noise Ratio
SPN	Sensor Pattern Noise
SVM	Support Vector Machine
